# When Cysts Strike: A Unique Case of Isolated Renal Hydatid Disease in a Child

**DOI:** 10.5146/tjpath.2025.13871

**Published:** 2026-05-30

**Authors:** Nikhil Kumar, Sandip Kumar Rahul, Harish Kumar Bohra, Vimal Singh Munda, Varsha Vijayan, Nidhi Priya Allie Barla

**Affiliations:** Department of Pathology/Lab Medicine, All India Institute of Medical Sciences, DEOGHAR, INDIA; Department of Pediatric Surgery, All India Institute of Medical Sciences, DEOGHAR, INDIA; Department of Microbiology, All India Institute of Medical Sciences, DEOGHAR, INDIA

**Keywords:** Hydatid cyst, Kidney, Pediatric

## Abstract

Echinococcus granulosus, also known as the dog tapeworm, causes echinococcosis or hydatid disease in humans. It is an anthropozoonotic and non-endemic disease. Hydatid cysts are most commonly found in the liver and lungs, but can occur in any other organ, including the brain, kidneys, bones, and peritoneal cavity. Isolated renal hydatidosis is an extremely rare condition, accounting for only 2-4% of all cases of hydatidosis, with its occurrence in children being even rarer. We are reporting a rare case of isolated renal hydatidosis in a 12-year-old boy.

## INTRODUCTION

Hydatid cyst disease, also known as echinococcosis, is an infection caused by Echinococcus granulosus, which typically affects dogs or other members of the Canidae family as the definitive host. The usual intermediate hosts of the larval stage (hydatid cyst) are sheep and other herbivores. Countries such as Australia, southern South America, and parts of the southwestern United States, where sheep are raised, are most affected by the disease. When a human inadvertently consumes E. granulosus eggs containing the hexacanth embryo, they become an intermediate host. Symptoms vary depending on the organ affected. While the lung is frequently involved in children, 70% of cysts form in the right lobe of the liver in adults. Unusual organ involvement includes the peritoneal cavity, spleen, kidneys, intestines, brain, bones, retroperitoneal space, abdominal wall, myocardium, and thoracic wall, with renal involvement occurring in only 2-4% of patients (1,2). Necrosis of surrounding tissue may result from the pressure exerted by a growing cyst. The symptoms depend on the location, size, and extent of the cyst. Patients may remain asymptomatic for a long period or present with nonspecific symptoms such as hematuria, lower abdominal or flank pain, or a palpable mass. Hydatiduria, a pathognomonic sign, occurs in 10-20% of patients and can cause colicky pain due to cyst rupture into the collecting system. Diagnosis is typically made through radiological examination, ultrasonography, or other imaging modalities such as computed tomography (CT) and nuclear magnetic resonance (NMR), in conjunction with specific serological tests. Protoscolices are usually identified after aspirating the cyst contents. The gold standard treatment is a combination of antiparasitic medications and surgical cystectomy.

## CASE PRESENTATION

**Figure 1:**
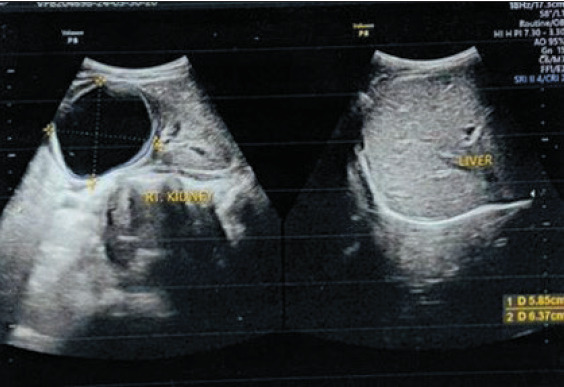
USG showing a large cortical cyst in the right kidney measuring 65 mm x 60 mm in size with uninvolved liver.

**Figure 2:**
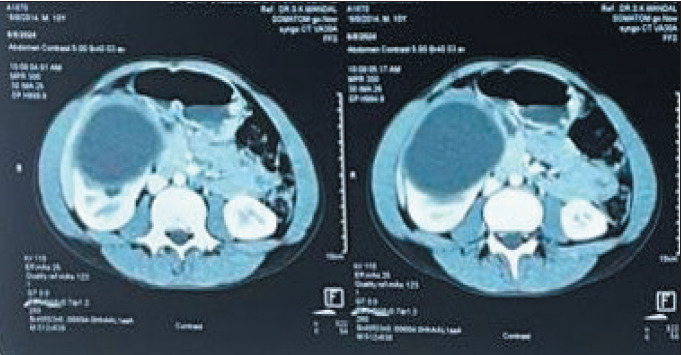
CT abdomen showing a cystic lesion in lower pole of the right kidney.

**Figure 3:**
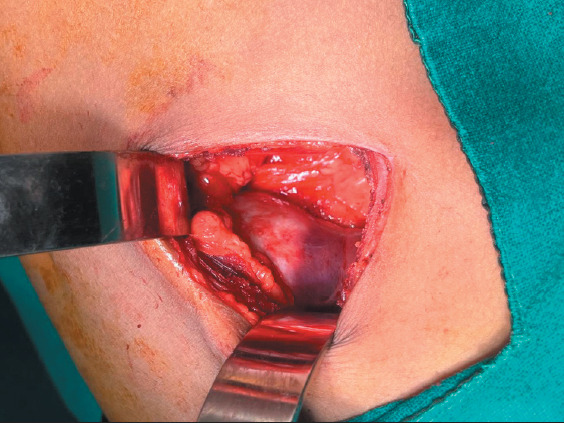
Removal of the cyst by upper transverse extraperitoneal approach.

A 12-year-old boy presented with the complaint of recurrent abdominal pain, which was intermittent and dull in nature, localized to the right lumbar and hypochondriac regions for the past three months with a history of close contact with dogs. On physical examination, a ballotable lump approximately 8 x 8 cm in size was found in the right hypochondrium, not moving with respiration. There was no hepatosplenomegaly. Ultrasonography of the whole abdomen revealed a large cortical cyst, approximately 6.5 x 6 cm, in the lower pole of the right kidney. Cortico-medullary differentiation was intact, and there were no masses, cysts, or abscesses in either lobe of the liver Figure 1. Based on these radiological findings, the following differential diagnoses were considered - simple renal cyst, renal hydatidosis, and necrosed renal tumor. A chest X-ray also showed no cystic masses. CT/MRI revealed a Bosniak II cyst in the lower pole of the right kidney Figure 2 which excluded the possibility of a necrosed renal tumor. A complete blood count showed eosinophilia (absolute eosinophil count was 700/μl) and mild anemia. The case was discussed with pediatric surgeons regarding the risk of intraoperative cyst rupture and dissemination that may result in anaphylaxis and recurrence. Surgery was planned, and complete removal of the cyst with pericystectomy was performed. The surrounding tissue was packed, and approximately 100 ml of fluid was aspirated intraoperatively to prevent spillage of cystic fluid Figure 3. Following aspiration, enucleation of the cyst contents was carried out. The cavity was washed with hypertonic saline and betadine, used as scolicidal agents. There was no communication with the pelvicalyceal system or ureter.

Grossing of specimen was done using personal protective equipment to prevent exposure. Sectioning of the cysts was done under formalin immersion to avoid aerosolization. The aspirated fluid was sent for cytological and microbiological analysis. Representative sections were taken from different areas of the cyst wall.

**Figure 4:**
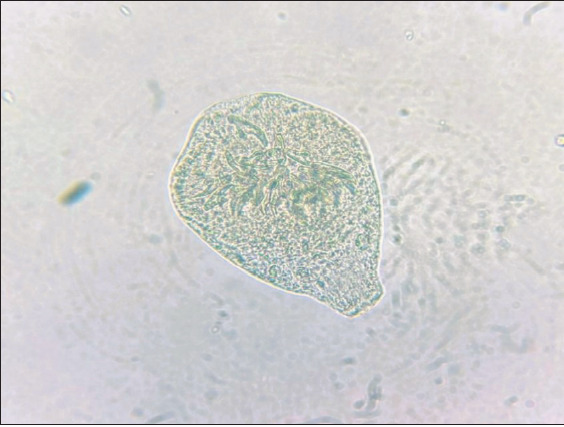
wet mount preparation of cyst fluid showing few scolices with refractive hooklets.

**Figure 5:**
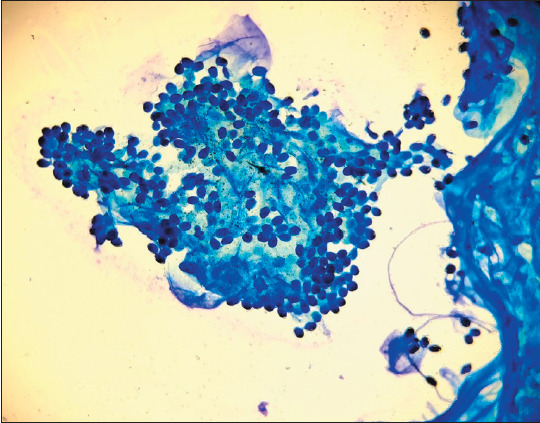
Protoscolices with armed rostellum with refractile hooklets of Echinococcus on wet mount smear of cyst content.

**Figure 6:**
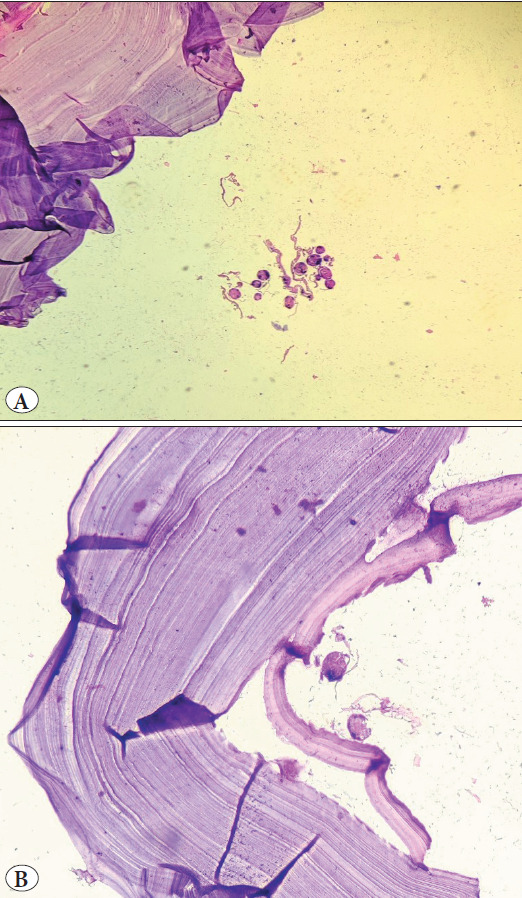
Protoscolices of Echinococcus, MGG X 40.A) Histology of the wall showed acellular lamellated layer and protoscolices, H&E X10. B) Histology of the wall showed acellular lamellated layer and protoscolices, H&E X10.

Microscopic examination of the wet mount preparation of the cyst fluid showed a few scolices with refractile hooklets Figure 4. MGG staining revealed numerous protoscolices Figure 5, and a histological examination of the cyst wall showed an acellular lamellated layer with protoscolices on H&E staining Figure 6.

## DISCUSSION

Hydatid cyst is an anthropozoonotic and non-endemic disease caused by Echinococcus granulosus, a parasitic tapeworm, and renal hydatidosis accounts for fewer than 2-4% of all hydatid cysts in humans. Although the liver and lungs are the primary organs affected by hydatid disease, the kidney is rarely involved, particularly in the pediatric patient. The identification and treatment of renal hydatid cysts in children are especially challenging due to their rarity. Renal hydatid cysts often present with clinical symptoms such as discomfort, fever, nausea, vomiting, bloating, anorexia, and weight loss (3).

Diagnosis of renal hydatid cysts is aided by radiological studies such as ultrasonography, followed by CT and MRI. According to the Gharbi et al classification system, hydatid cyst disease can be classified into 5 classes. Type I is well defined anechoic cyst with thickened wall, type II displays detachment of the germinative membranes, type III includes multicystic multi septated lesions, type IV shows heterogenous degenerated cyst with internal echoes, and type IV involves calcification (4). A CT scan provides more accurate results than ultrasonography (5). Since ELISA and serological testing are positive in only 50% of cases, they can offer limited assistance. The indirect hemagglutination test is more effective in detecting renal hydatid cysts (6). However, the sensitivity of these tests drops to around 25-56% in extrahepatic disease, limiting the usefulness of serology as a diagnostic tool (7).

Hydatid cyst should always be considered as a differential diagnosis for renal cysts, particularly in endemic areas. Surgical excision with pericystectomy or partial/total nephrectomy is the treatment of choice, depending on the extent of renal parenchymal damage. In our case, the renal parenchyma was not involved and so only enucleation of the cyst contents was performed, using hypertonic saline and betadine as scolicidal agents, and oral albendazole was prescribed for one month.

## CONCLUSION

Renal hydatid cysts are rare, and they are even rarer in the pediatric age group. Hydatid disease should always be considered as a differential diagnosis in a child with a cystic renal mass, especially in endemic areas. Renal hydatid disease may sometimes mimic renal calculi and can be missed radiologically. Surgical management, combined with perioperative anthelmintic drugs, is the treatment of choice in most cases. Follow-up is also crucial to detect any recurrences.

## Conflict of Interest

The authors declare that they have no conflict of interest.
